# "It is the scourge of God" Misconceptions of COVID 19 Pandemic Among Older Market Traders In Ibadan, Nigeria

**DOI:** 10.1093/geroni/igab046.3206

**Published:** 2021-12-17

**Authors:** Olubukola Omobowale, Adeola Fowotade

**Affiliations:** 1 University of Ibadan, Nigeria, Ibadan, Oyo, Nigeria; 2 College of Medicine,, Ibadan, Oyo, Nigeria

## Abstract

Abstract Background: Community perception on COVID-19 can influence the development of the right attitude towards mitigating the spread of the Sars CoV 2 virus. Older adults are at risk of severe infections and mortality is high among them. Objectives: This study was conducted to document the knowledge, perceptions and misconceptions of COVID-19 among older market traders in Ibadan, Oyo State, Nigeria. Methods: A cross-sectional study conducted in two densely populated markets in Ibadan. An interviewer-administered semi-structured questionnaire was used to collect data on the knowledge and perception of COVID-19. Data were analyzed using SPSS version 23. Level of significance was set at p<0.05. Results: A total of 321 respondents were sampled. All participants were aware, source was mainly through radio (93.5%), and 65.8% believed COVID-19 was as a scourge from God for punishments of sins. Only 41.1% had good knowledge of spread with personal contact (95.3%) mostly reported. On knowledge of symptoms and preventive measures, dry cough (84.7%) and frequent hand washing (95.6%) were mostly reported. Knowledge of cause was significantly associated with age (p=0.04) and marital status (p=0.001), while level of education (p=0.012) was significantly associated with knowledge of spread. Conclusion: Misconceptions about the knowledge of the cause and spread of COVID-19 were prevalent among the study population. The implication of this finding among older adults and the significant effect of some sociodemographic factors on the knowledge of the cause and spread of COVID-19 calls for urgent health-promoting interventions that would dispel the misconceptions.

